# Supplementary material for: Molecular Subtyping Resource: a user-friendly tool for rapid biological discovery from transcriptional data
Source: Dis Model Mech. 2022 Mar 30;15(3):dmm049257. doi: 10.1242/dmm.049257 (PMC8990914; doi:10.1242/dmm.049257)
Supplement: Supplementary information [file dmm-15-049257-s1.pdf]

**Dataset 1.**

Click here to download Dataset 1

**Dataset 2.**

Click here to download Dataset 2

Disease Models & Mechanisms • Supplementary information
